# Engineering Protocells: Prospects for Self-Assembly and Nanoscale Production-Lines

**DOI:** 10.3390/life5021019

**Published:** 2015-03-25

**Authors:** David M. Miller, Jacqueline M. Gulbis

**Affiliations:** 1 The Walter and Eliza Hall Institute of Medical Research, Parkville VIC 3052, Australia; E-Mail: jgulbis@wehi.edu.au; 2 Department of Medical Biology, The University of Melbourne, Parkville VIC 3052, Australia

**Keywords:** protocells, microfluidics, self-assembly, synthetic biology

## Abstract

The increasing ease of producing nucleic acids and proteins to specification offers potential for design and fabrication of artificial synthetic “organisms” with a myriad of possible capabilities. The prospects for these synthetic organisms are significant, with potential applications in diverse fields including synthesis of pharmaceuticals, sources of renewable fuel and environmental cleanup. Until now, artificial cell technology has been largely restricted to the modification and metabolic engineering of living unicellular organisms. This review discusses emerging possibilities for developing synthetic protocell “machines” assembled entirely from individual biological components. We describe a host of recent technological advances that could potentially be harnessed in design and construction of synthetic protocells, some of which have already been utilized toward these ends. More elaborate designs include options for building self-assembling machines by incorporating cellular transport and assembly machinery. We also discuss production in miniature, using microfluidic production lines. While there are still many unknowns in the design, engineering and optimization of protocells, current technologies are now tantalizingly close to the capabilities required to build the first prototype protocells with potential real-world applications.

## 1. Introduction

Advances in recombinant molecular biology in the 1990s heralded new approaches enabling exploration of biological systems in unprecedented detail. The ability to clone, modify and express a vast array of cellular proteins was extended yet further by the capacity to reconstitute recombinant macromolecules into simple artificial systems. The resulting explosion of knowledge has yielded a far deeper understanding of fundamental processes underscoring cellular biology. Science is fast approaching the point where such information can be harnessed and used in the design and generation of “protocells”, rudimentary synthetic organisms inspired by biology. Carefully selected components of metabolic pathways can, in theory, be utilized to produce purpose-built protocells with highly specific functions. The potential applications are significant and varied; for example in environmental cleanup, biofuel production, and medical and pharmaceutical devices for drug production and drug delivery.

At present, designer organisms are based on living cells that have undergone modifications to selected metabolic pathways or have had new pathways incorporated. This type of tailoring for desirable characteristics can be thought of as a “top down” approach to design. Amongst the most impressive examples to date are the generation of usable fuels by modification of both yeast and bacteria, including direct synthesis of alkanes from free fatty acids by introduction of the key enzymes fatty acid reductase and aldehyde decarbonylase [1]. Others include the production of alcohols with examples of the extensive metabolic modification to produce isobutanol [2,3,4,5,6], butanol [2,7], propanol [2], isopropanol [8] and 3-methyl-1-butanol [9] as fuel components. Impressively the production of some of these fuel molecules has even been linked to CO_2_ fixation in photosynthetic organisms [10,11]. There are also instances of pharmaceutical precursors being successfully produced in microorganisms by introduction of biosynthetic pathways, lowering the potential cost of drug manufacture. These include precursors of the antimalarial artemisinin produced in *E. coli* [12] or a modified yeast strain [13,14], and of the chemotherapeutic *taxol* in *E. coli* [15].

The simplicity of modifying existing organisms in this fashion is not without appeal. “Housekeeping” metabolic processes, including the ability to generate energy currency, are already present within host organisms and their endogenous biosynthetic pathways can be harnessed to produce precursors for synthetic chemistry. Despite these obvious benefits there are some inherent limitations. For instance, altering an organism’s metabolism decreases its overall fitness, as a result of the build up of inhibitory precursors [16,17,18] or from the inherent toxicity of the preferred end products to the host cell. For example yeast cells engineered to produce artemisinic acid, a chemical precursor to artemisinin, are characterized by a marked increase in indicators of cellular stress responses [19]. In such instances “protocells” could offer a superior alternative. Designed instead from the bottom upwards, they comprise elementary systems containing only a minimal complement of components required to execute their desired function(s). The significant design potential of protocells has advantages over top down approaches that could prove particularly important in the design of medical devices, for example, where biocompatibility could be engineered from first principles.

Theoretically, the degree of control over protocell design far exceeds that which can be obtained by re-directing living cells, which still holds many unknowns. While optimization can be accomplished to some extent through metabolic engineering of existing organisms, e.g., by evolution and selection [20] host organism modification for product production is not trivial (discussed in [21]). By cherry-picking key metabolic pathways or enzyme cascades and omitting non-essential components, protocell performance can be optimized and many of the problems associated with modifying existing organisms can be avoided. In simpler protocells, protective cellular pathways can be eliminated and resistance to product toxicity engineered—for instance by exclusion of target molecules. The formation of unwanted byproducts can be minimized by avoidance of competing pathways. In the absence of the cellular components responsible for degrading a product of interest, yields can be improved and efficiency maximized. This level of control comes at a cost. To date the development of true protocells has been hindered by the significant technical challenges involved, largely due to the difficulty in combining individual components of diverse cellular origins into a self-maintaining and functionally competent system. The high inherent complexity of living cells, with multiple interconnected pathways and complex regulatory processes, is a consequence of their evolution over millennia by a process of natural selection. A complete genome equips them to replicate, service internal energy needs and exist independently. The very simplicity that advantages protocells could also be their downfall, in that *in vitro* assembly requires that all essential components be provided. At a minimum, this includes a collection of enzymatic components required for energy generation and product synthesis. Despite these hurdles, many of the technologies required to build protocells are now accessible. Recent studies have combined promising design with incorporation of relatively complex protein machinery into liposomal compartments—the membrane-bound capsules that isolate the working components from external factors just as living cells compartmentalize themselves with lipid membranes.

This article provides an overview of protocell technology, moving from what is currently known to suggesting how novel application of existing methodologies could be utilized as groundwork to construct complex artificial systems. The first section discusses building simple prototype protocell machinery from individual components. The second is a future perspective on self-assembling protocells—which represent a significant leap in complexity. One might envisage protocells with a limited “genome” of nucleic acids and the machinery required for maintenance, repair and auto-production of specific proteins—all controlled by systems that sense and respond to external triggers. A third section presents possibilities for automating protocell production. The review finishes with a selection of points for contemplation and consideration. While there are boundless possibilities, the designs considered here follow a classical template of a lipid membrane-bound compartment in which proteins and/or nucleic acids are incorporated. The challenges facing the field of synthetic biology are substantial but not insurmountable and the potential of this exciting and emergent frontier area remains to be realized.

## 2. Protocells—From the Ground Up

Modeling protocells after biological cells has obvious advantages; most notably that extensive cataloguing of proteins and nucleic acids by genome projects has yielded databases of potential protocell components. The core componentry can thus be derived from known proteins or ribozymes, starting with those that have proved functional from studies *in vitro*. A classical living-cell design can be deconstructed into component systems for “life” and “function” (e.g., fuel synthesis or pharmaceutical production). Whilst the processes to support life are essential for biological cells, many of these systems can be omitted or greatly simplified in protocell design, and pathways relating to function can be tailored to optimize performance. 

**Figure 1:**
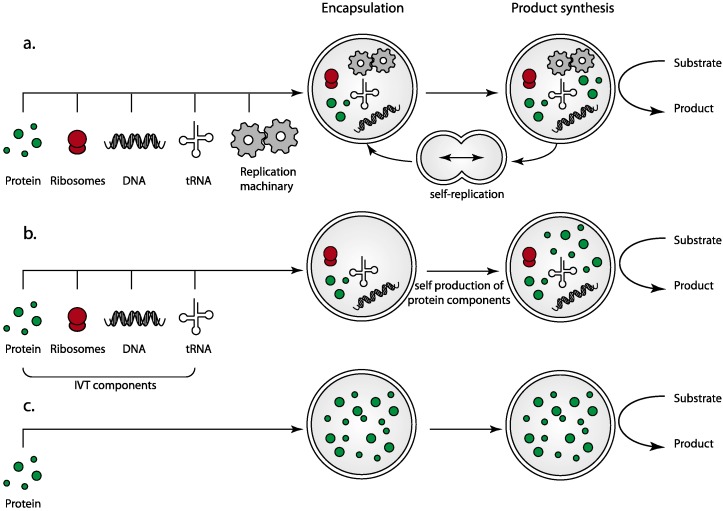
Simplified overview of the designs of lipid encapsulated protocells. (**a**) A fully replicating and autonomous protocell where all components for metabolism, product synthesis, maintenance and replication are provided or are readily produced by *in vitro* protein synthesis; (**b**) a simplified protocell that cannot replicate, but is capable of producing an entire metabolic pathway including receptors for sensing and responding to environmental cues by protein production from transcription/translation of provided nucleic acid templates (*in vitro* transcription/translation—IVT); and (**c**) further simplification of the design results in elimination of the transcriptional machinery components with all components required for the protocell metabolism added during protocell production. All three designs may still retain equivalent metabolism, that is, they all may produce the same end products, but the simplified designs have a significantly reduced component count.

Using biological cells as inspiration for protocell design, it is theoretically possible to generate protocells spanning a range of complexities (Figure 1). At the most complex end of the spectrum, designs would result in autonomous entities, incorporating not only the machinery for product synthesis and energy generation, but also genomes, protein transcription and translation machinery and even the capacity for replication and cell division to create a true artificial life form capable of self-renewal (Figure 1a). More simplified designs dispense with the replication capacity but could retain some elements of genomic information and protein transcription and translation machinery, allowing for renewal of individual components and creating a protocell with an element of autonomy but without the ability to replicate (Figure 1b). In its simplest form, the protocell would consist only of the required molecular machinery to perform its prescribed role encapsulated within an isolating membrane (Figure 1c).

Even with this low level of complexity, protocells may perform a specified function as competently as biological cells (or more highly engineered protocells). The downside is that such simple protocells are prone to degradation and deterioration of components, and the absence of cellular repair mechanisms makes them relatively short-lived and needing continual replenishment. Longer lifetimes would require inbuilt repair and maintenance, which adds complexity. Even the most primitive of needs, the replacement of component proteins, requires a simple genome encoding the proteins and provision of transcription and translational machinery (or simply translational machinery if the genome is manufactured of RNA) for synthesizing them. This not only necessitates a method for regulation of protein production from the provided genome, to ensure all components are produced to required levels at appropriate times (discussed further in Section 5.6), but also a complex system for the production of substrate ribonucleotides (for RNA production) and amino acids (for protein synthesis) and energy. At the very least a method for providing all required substrates to the protocell is needed.

### 2.1. The Complexity of Replication and Cell Division: The Benefits of Simplification

Simple protocells as described above are autonomous, designed to function as a self-contained entity, but exclusion of the capacity to self-replicate cannot be considered a “life” form. There are advantages and disadvantages to incorporating the ability to replicate, and while this would ensure a perpetually functioning system it would also necessitate a serious jump in design complexity. For instance, it would require inclusion of biosynthetic circuits for the manufacture of every component (lipids, proteins, nucleic acids and additional small molecules). Far more dauntingly, a mechanism for protocell division and component distribution would have to be devised and implemented. For successful division, the encapsulating membrane must increase in size and then split evenly without loss of internal components. Component partitioning from mother to daughter cells *in vivo* is a very tightly controlled process and an analogous mechanism for redistribution of internal components to daughter protocells is a particularly challenging prospect. At this juncture, our knowledge of cellular componentry is inadequate to accommodate all the requirements. Thus, while protocell division has recently been discussed (see reviews by [22,23]) and some studies have reported systems utilizing biological parts that mimic aspects of replication (for example see [24]), this review will focus on more tangible technologies that can be used to assemble protocells from component parts rather than on production of a “living” and replicating synthetic super-protocell. 

In short, while a replicating protocell would be an impressive achievement, a “stripped-down” design with equivalent metabolic functionality is a more tractable proposition, given the current status of scientific knowledge and technology. Reduction of component number has the advantages that there are fewer “unknowns” to overcome and biocompatibility and biodegradability are more easily engineered. Simplified protocell “machines” could potentially be tailored to maintain metabolism for a predetermined or limited period of time followed by degradation and clearance. This could be included as a default in the design of protocells for medical drug delivery or environmental cleanup. In addition to allowing for simplified designs, engineering a finite lifespan would eradicate ethical concerns regarding uncontrolled release of synthetic organisms.

### 2.2. Protein-Only Protocells

A “protein-only” protocell represents the simplest protocell design, constructed entirely of constituent proteins in aqueous media protected by a simple lipid membrane. A judicious choice of componentry to minimize biological requirements can be coupled with limited capabilities for self-diagnosis and repair. In metabolic protocells, product synthesis or substrate breakdown is contingent upon a suitably engineered metabolic pathway, replete with catalytic enzymes to perform the protocell function. Auxiliary metabolic pathways must also be present to support the primary processes, e.g., for precursor production from provided chemical feedstocks. These are the primary considerations for protocell design. They additionally require means of energy conversion to produce the chemical energy essential to function, similar to the reactions performed by molecular machinery within mitochondria and chloroplasts of living cells.

Integral membrane proteins traversing the bounding membrane may be required to allow for transport of small molecules, feedstocks and products into and out of the protocell to support metabolic reactions. The folding, and therefore function, of membrane proteins is known to be heavily influenced by the biophysical properties of the membrane in which they are embedded [25,26,27,28,29,30,31], which are in turn determined by the lipid composition of the membrane [32]. The choice of protocell membrane is key, not simply to generate a boundary to contain internal machinery but also to provide an environment appropriate for membrane proteins integrated into the protocell surface to function.

Precursors to protocells of this ilk have been demonstrated in the form of very simple systems comprising enzymes and enzyme pathways incorporated into liposomes (for example see [33]). As early as 1970, the most basic protocell precursors were developed, initially by encapsulating the enzyme lysozyme within liposomes [34]. Later work demonstrated the encapsulation of multiple enzymes, such as the incorporation of glucose oxidase, horseradish peroxidase and lactoperoxidase into liposomes to generate a simple functional enzyme cascade with measurable production of hydrogen peroxide in response to the addition of d-glucose to the external medium [35]. Studies such as these have paved the way towards simple protocell production, and protein and lipid only designs are now within reach*.* Not only are they relatively simple to devise and apply, modular elements with differing functions can be incorporated to generate more complex protocells, increasing the range potential applications whilst maintaining simplicity of design. Construction of protocells *de novo* could be achieved by utilizing biological components that promote self-assembly or by building miniature production lines based on microfluidic technology, as discussed in later sections. 

### 2.3. The Protocell Membrane and Encapsulation of Internal Components

A major challenge in protocell design is developing a bounding membrane that efficiently encapsulates the internal components whilst allowing selective ingress and egress of precursor and product molecules via transporter proteins and pores traversing the membrane. Both naturally occurring lipids and synthetic lipid mimetics can assemble into membranes qualitatively similar to cell membranes, with the additional benefits of the ability to be tailored during manufacture to control their properties. They offer superior biological compatibility and, unlike many artificial polymer membranes, are biodegradable.

The minimum size requirement for artificial cells, set by a requisite concentration of individual components, is surprisingly small (200 nm) for simple protein synthesis machines [36]. It would be expected to increase with internal complexity to maintain component concentrations at an acceptable level, although perhaps to a lesser extent than expected due to spontaneous molecular crowding effects (as discussed in [37]). Suitable methods for producing artificial liposomes of this size are well established. However despite numerous examples, the efficient encapsulation of internal components required for protocells has only recently been described. Unilamellar liposomes or vesicles suitable for protocell systems are typically referred to as large unilamellar vesicles (LUVs), which have diameters less than 1 μm, and giant unilamellar vesicles (GUVs) with diameters in the micron range. Based on combinations of biological phospholipids similar to cell membranes, both serve as vehicles for the physicochemical analysis of embedded integral membrane proteins. Preparation of LUVs normally utilizes extrusion methods [38] whereas GUVs have traditionally been produced by budding and release of the giant liposomes from swollen lipid films [39,40] or electroswelling [41]. Thus while LUVs are of uniform, reproducible, size, GUVs have a broad size distribution, even within individual batches.

**Figure 2:**
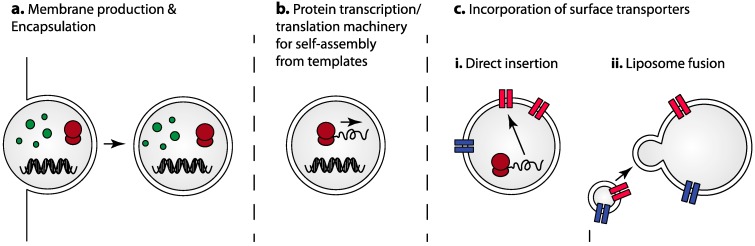
Component parts and strategies for self-assembly. The minimal steps required for the *in vitro* construction of protocells that self-assemble, or partially self-assemble, from component parts would consist of: (**a**) the production of the protein and nucleic acid components using traditional protein purification strategies, followed by encapsulation within the protocell membrane; (**b**) the utilization of protein transcription/translation machinery for building individual protocell protein components from nucleic acid templates creating a self-assembling protocell; and (**c**) incorporation of surface transporters and receptors for sensing and transport either (i) directly from *in vitro* transcription/translation; or (ii) through liposome fusion where membrane proteins already incorporated in liposomes are fused with the protocell membrane. The steps shown are not mutually exclusive in the process of protocell production.

Liposomes formed by these production methods have successfully been employed for reconstitution of enzyme activity and enzyme biosynthetic pathways [42,43,44], cytoskeletal protein components [45], simple genetic networks [46] and notable physiological examples of enzymes for lipid biosynthesis [47,48,49] where changes in the lipid constitution of the vesicle membrane provide the activity readout. The downside is that encapsulation efficiency is low as a result of the relatively large unencapsulated volumes unless the internal components can be directly injected into GUVs [47,50]; this would be infeasible for large-scale production. A high encapsulation efficiency of the internal components can be realized utilizing lipid monolayer stabilized emulsions as a starting material (or “inner” monolayer) followed by formation of the outer lipid monolayer to create a complete bilayer encapsulating the internal components. The outer leaflet of the membrane can be formed directly by passing the lipid monolayer stabilized emulsion from an oil to aqueous phase with a lipid monolayer at the interface (Figure 2a), resulting in a population of liposomes that is almost 100% unilamellar [51]. This technique, originally described in [52] and developed by several groups since [53,54] has formed the basis of some of the impressive examples of encapsulation of protein and enzymatic machinery in liposomes including assisted membrane protein expression [55], compartmentalized artificial synthetic enzyme pathways [56], and controlled assembly of a membrane-associated cytoskeleton [57].

## 3. Self-Assembling Protocells—The Next Step

Living cells maintain their function and integrity through mechanisms that are staggering in their complexity, involving thousands of individual proteins assembled into larger complexes and hundreds of intricate molecular networks and pathways. Although it is not yet possible to replicate the complex nested processes defining living cells in an artificial system, a functioning protocell does not require everything a cell does. Existing knowledge and molecular technologies can be harnessed to select and engineer individual components for incorporation into protocell designs, and there is potential to use existing molecular processes as inspiration for engineering self-assembly of simple protocells. Self-assembling protocells could be instilled with the ability to generate selected internal components autonomously by provision of a simple genome and machinery, using means not altogether distinct from *in vitro* protein synthesis experiments (for examples of this design see [36,55,58,59,60]). While current technologies are not advanced to a point where complete self-assembling protocells can be created, it is possible to identify an array of potential precursors and strategies for self-assembly, and through careful design some non-essential factors may be eliminated and others provided by alternate routes.

The physical properties of many biological components themselves promote self-assembly into larger complexes and higher order structures. One key example is the encapsulating membrane, an essential element of a protocell. In living cells this is a lipid bilayer where the polar head groups face into the solution and the hydrocarbon tails form an essentially impermeable core. In an aqueous environment, many lipids naturally aggregate into a bilayer and can be prepared as single or multilayered capsules of uniform size. From this nucleus, a multitude of approaches to design of a simple, self-assembling protocell are possible. Lipid-stabilized emulsions dispensed in regulated fashion are a practical means of automating membrane formation, while controlled fusion of appropriate proteoliposomes could be used to automatically incorporate recombinantly produced molecular transporters and channels. Provision of transcription/translation machinery for protein synthesis and a miniature genome encoding essential proteins would render a degree of self-sufficiency. A continual supply of amino acids and nucleic acid building blocks could be ensured via incorporated membrane protein pores. Much of this has already been achieved in simpler systems.

### 3.1. Self-Assembly Utilizing Transcription/Translation Machinery for Protein Synthesis

From a self-assembly perspective, some of the most exciting possibilities include protocells in which protein components are translated within the protocell from a nucleic acid template (Figure 2b). These would require encapsulation of complete suites of enzymatic machinery for protein expression from nucleic acids (*in vitro* transcription/translation). Liposomal protein-synthesis machines have been produced consistently, with iterative improvements in efficiency and complexity starting from basic protein synthesis machines [46,58,59,61], to those that produce more complex enzymatic pathways [48] and simple membrane proteins [55,60]. Some of the described designs are of impressive complexity, incorporating 31 individually purified protein components, ribosomes, tRNAs, nucleotide triphosphates and several other small molecules as the basis for protein expression [62]. While not yet exploited in the construction of directed protocell machines, these examples represent significant progress toward such a goal and offer a tantalizing vision of the opportunities ahead.

### 3.2. Incorporation of Surface Transporters and Receptors

In living cells, qualities such as sensing, communication, metabolite uptake and product release are conferred by integral membrane proteins in the form of surface receptors, ion channels and molecular transporters. Similar functions are required in protocells and these classes of proteins offer a plethora of options for incorporation into protocell design and assembly, with the proviso that reliable methods of production and efficient reconstitution into protocell membranes are developed. 

Integral membrane proteins are still regarded as complicated in terms of their biochemistry and their folding and function is reliant on an optimal membrane environment, making them challenging to study in their native state. As a consequence of the technical difficulties, there are relatively few examples of well characterized membrane proteins and techniques for generating functional recombinant membrane proteins have been slower to develop than those well publicized for more soluble cytosolic or secreted proteins. Progress in the area is fast however, and the once seemingly insurmountable hurdles to producing membrane proteins by recombinant methods are tumbling. A recent push toward studying membrane protein function in a native membrane environment under controlled conditions has resulted in the development of effective methods for incorporation of recombinant proteins into lipid membranes *in vitro* [63]. These same techniques could be adapted to the incorporation of surface transporters and receptors into protocell membranes. However any method for efficient integration of membrane proteins must also be compatible with reproducible assembly of the protocell interior. Several membrane proteins have been successfully incorporated into GUVs of a size suitable for a protocell, including potassium channels [64], mechanosensitive channels [65], the protein translocase SecYEG [66], a Ca^2+^ ATPase and bacteriorhodopsin [67]. Whilst these approaches have been successful, the electroswelling process that typically underpins the experiments results in inefficient encapsulation of internal components and hence is unsuitable for protocell production.

Possible approaches to efficient incorporation of both membrane proteins and encapsulation of internal components could entail direct production of membrane proteins by *in vitro* transcription/translation methods, followed by direct insertion of internally expressed proteins into the membrane [48,61] (Figure 2c). Other options include assisted insertion utilizing translocation machinery from extracts of cellular endoplasmic reticulum (ER microsomes) [55], and incorporation of externally expressed membrane proteins [68]. This has been demonstrated in an impressive example of *in vitro* transcription/translation of the individual components of the SecYEG translocon, followed by spontaneous insertion of the functional translocase into liposomes and the subsequent translocation of *in vitro* synthesized substrate proteins into the liposome interior [69]. On the other hand, as incorporation of purified membrane proteins into sub-micron liposomes is readily achievable in many cases, an alternative is to fuse small liposomes containing the required membrane proteins with custom “protocell GUVs” containing prescribed internal components, greatly simplifying the assembly process. Possibilities for liposome fusion include the use of synthetic peptide “fusogens” incorporated into the membrane [70,71,72,73], or simple chemical methods for liposome fusion [74,75,76]. Both appear facile to implement and chemical methods in particular may be helpful in decreasing the number of protein components required. Membrane proteins have been functionally reconstituted into GUV membranes in this way, for example the incorporation of bacteriorhodopsin from small liposomes using the fusogen peptide WAE [70]. 

### 3.3. Membrane Pores for Incorporation of Internal Protocell Components

In addition to sensing and communication, specific membrane proteins incorporated into the protocell surface could potentially be used as vehicles for self-assembly. These might include non-specific “controllable” pores that allow only small molecules to pass through the membrane or macromolecular transport machinery that specifically permeabilizes the membrane to nucleic acids or proteins. It broaches the possibility that a protocell could be constructed from a liposome shell with a molecular transport system in the membrane, to which externally added components “self-assemble” into a complete protocell. This has the advantage of incorporating flexibility into the componentry of a basic system.

### 3.4. Engineered Protein Pores for Incorporation of Small Molecules into Protocells

**Figure 3:**
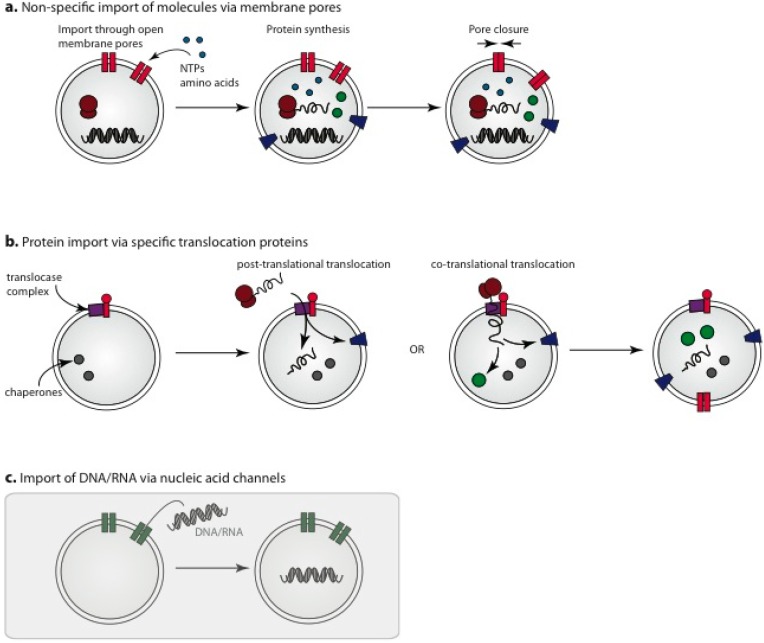
Assembly methods for protocells utilizing incorporated membrane protein channels and pores for the provision of internal components. (**a**) All components required for protein synthesis, by *in vitro* transcription/translation from an artificial nucleic acid genome, are encapsulated into the protocell precursor with all small molecules provided through integrated non-specific gated membrane channels that permeabilize the protocell membrane. Small molecules are continually supplied until the completion of protocell self-assembly, at which point the protocell is sealed from the external environment by controlled pore closure; (**b**) Additional possibilities for utilizing membrane protein pores include the use of organelle derived protein tranlocase components that allow for either the post-translational or co-translational import of polypeptides; and (**c**) nucleic acid channels to provide required nucleic acid components from the external solution. Strategies within the grey-boxed area have not yet been thoroughly explored in *in vitro* systems.

Nonspecific membrane pores have potential in protocell design if their opening and closing could be controlled to allow ingress of precursor molecules into an empty protocell. Such channels could be used in protocells to replenish substrates or to streamline self-assembly allowing small molecules to enter during the construction phase, but preventing the release of internal macromolecules. Haemolysin is an example of a nonspecific membrane pore where opening and closing might be controllable. Evidence for external control has been provided in the form of an engineered variant that gates in response to an externally applied chemical stimulus [77]. Haemolysins are not unique in this sense, a non-specific mechanosensitive channel (MscL) has been engineered to open in response to a light stimulus [78] or can be indirectly controlled by manipulation of the physical properties of the surrounding lipid [65]. Additionally there are possibilities for the inclusion of artificial, non-protein based, pores that are controllable by pH changes, for example [79]. Finessing modes of control appears feasible in the medium term and there is even potential to produce some channels directly within protocells, as *in vitro* transcription/translation of membrane pores in a liposome-encapsulated system has been demonstrated [60]. The utility of non-specific membrane pores to enable substrate entry into liposomes for catalysis by encapsulated enzymes [80], or to provide reagents necessary for spontaneous polymerization and assembly of internal components (for example cytoskeleton [51,57]) has also already been shown. In another particularly pertinent example, α-haemolysin incorporated into liposomes allowed amino acids and nucleotide-triphosphates to be supplied for production of a target protein by *in vitro* transcription/translation [61], essentially forming a protocell precursor. This design could be reworked to establish essential enzymatic pathways during generation of protocells, completing the construction process with a controlled pore closure to seal the system (Figure 3a).

### 3.5. Import of Pre-Assembled Protein Components

Protocell design is still in the early stages. There are possibilities for harnessing biological components for assembly that have not yet been utilized, in particular protein translocation machinery for the directed import of externally synthesized proteins. Translocases are dynamic multicomponent macromolecular assemblies that associate closely with cellular chaperones with the function to transport proteins unidirectionally across membranes during (co-translational) or immediately following (post-translational) ribosomal protein synthesis. These systems are of particular interest, as a major function of translocases *in vivo* is the controlled incorporation of thousands of different proteins into the organelles of mitochondria and chloroplasts (via the TIM/TOM and TIC/TOC complexes, respectively) essentially resulting in self-assembly of these organelles from externally added components (for reviews see [81,82,83,84,85,86,87]). 

While translocation and folding have extremely complicated biology, much of which is only partially understood, some encouraging preliminary results from production, purification and functional reconstitution of translocases into *in vitro* membrane systems have been reported. The complete translocase complexes are overly complicated for protocell construction, facilitating import of proteins across double membranes *in vivo*, whereas current designs for protocells have only a single membrane envelope. This simplifies protein translocation, reducing the complexity of the translocation components required and opening up avenues for simplified complexes to be harnessed for the incorporation of proteins into protocells (Figure 3b). While the *in vitro* reconstitution of an entire functional translocase system has not yet been achieved, evidence suggests controlled translocation across a single membrane may be possible. A core complex of the outer membrane TOM purified from *Neurospora crassa* is able to translocate inner-membrane bound protein precursors in liposomes containing specific chaperone components [88]. The import of soluble proteins may require additional components for an ATP driven chaperone motor [89,90,91,92]. Another positive example is Tim22, a core component of the TIM complex of the mitochondrial inner membrane. Purified Tim22 exhibits channel-like voltage-sensitive conductance in an *in vitro* system [93], and with inclusion of additional components accumulates precursor proteins at intermediate stages of import [94]. The TIC and TOC translocases of chloroplasts have similarly been shown to be functional *in vitro* to a limited extent. A purified TOC complex has been shown to form a functional channel in liposomes using GTP as an energy source [95] and the import pore of the TIC family has also been reconstituted in an artificial liposome system [96,97].

The Sec complex is a distinct “translocon” system that is a major player in the secretory system of eukaryotic and prokaryotic cells. This has been produced and reconstituted *in vitro* culminating in transport of model proteins into artificial liposomes [98,99,100]. The accessory insertase YidC has also been shown to incorporate membrane proteins into a functional state in liposomes [99,101].

An entire translocase system built from minimal components has not been demonstrated and the import of entire proteins into developing protocells is likely to be problematic for a number of reasons. Import is known to be an active process, although the full details have not yet been elucidated. Even if it could be tailored to passive import, high external protein concentrations would be required to promote diffusion of viable concentrations into the protocell. Import is also specific, requiring the inclusion of signal peptides for selective recognition by the import machinery, and further significant engineering of the target proteins would be required so that they remain import competent. In similar vein, import is likely to require ancillary chaperones. However, if protein pores could be harnessed, even for a subset of the required protein components, it would enable external addition of protein components of desired metabolic pathways for preassembly in empty protocells. This would add significantly to the ground that can be covered and will in some cases be a preferable option to intrinsic component synthesis.

### 3.6. Nucleic Acid Channels

In addition to the import of proteins into self-assembling protocells, the import of nucleic acid fragments into the protocell may be desirable, for instance if inclusion of these in the original liposome preparation presents incompatibilities. Although not yet fully investigated *in vitro*, nucleic acid channels in protocell membranes could potentially be employed to permit selective entry of nucleic acid molecules into protocells during the construction phase (Figure 3c). While there is not a great deal of information available, assessments of nucleic acid channels (e.g., NACh) show that function is retained *in vitro* [102,103]. The dsRNA channel SID1 is another candidate capable of permeabilizing membranes to RNA hundreds of base pairs long [104], although *in vitro* reconstitution and functionality remains to be demonstrated. More complex nucleic acid structures could possibly be assembled from shorter imported fragments within the protocell, e.g., for the production of scaffold components.

### 3.7. Segregation of Components: Engineered Scaffolds and Compartmentalization

In even the simplest protocell design, multiple metabolic pathways will be required to be present and operate at acceptable levels of efficiency. There are advantages to segregating these different chemical processes. It can increase efficiency by keeping the reagents for a particular process in one place or it can sequester toxic by-products to preserve other parts of the system. Living cells achieve this by a variety of means, including compartmentalization (mitochondria, nucleus, peroxisomes), co-assembly of components that act together into large complexes [105] or simply co-localization to cellular membranes [106]. In simple protocells, protein components arise either by encapsulation or synthesis by *in vitro* transcription/translation and are stochastically dispersed throughout the entire space. This section explores two possibilities for improving protocell efficiency and segregating processes—scaffolding and compartmentalization. 

**Figure 4:**
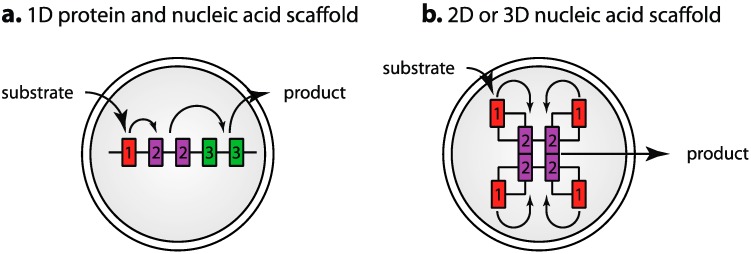
Assembly of metabolic pathway components on scaffolds for enhancement of pathway activity. The engineered scaffolds contain specific binding sites to allow the individual components to be localized to the scaffold. Current designs consist of either (**a**) assembly of metabolic pathway components on simple 1-dimensional nucleic acid or protein scaffolds where the stoichiometry of components can be adjusted by scaffold design for optimal pathway efficiency and product production; or (**b**) more complex designs for self assembly of components in multidimensional arrays as a result of the engineered scaffold’s ability to self assemble into more complex structures. Examples have been demonstrated utilizing both RNA and DNA as the scaffolding material.

Engineered scaffolds can improve catalytic efficiency by assembling individual enzyme components into artificial metabolic complexes (Figure 4). This has been recently reviewed [107,108] and could be readily implemented in protocell design. Some interesting ideas have come from elaborate designs, where artificial DNA [109,110,111], RNA [112,113] and engineered protein scaffolds [114,115] were used to constrain a cluster of enzymes spatially, either in 1 dimension (Figure 4a) or higher order 2- and 3-dimensional structures (Figure 4b), resulting in enhanced or altered activity of the metabolic or signalling pathway of interest. The numerous recent examples of these designs and the success with which they have been implemented with vastly different scaffolds indicates this could be a very successful strategy for metabolic optimization. 

Scaffolding may also be of use in ensuring that suitable stoichiometric ratios of the different enzymatic components are added to each protocell for optimal catalytic efficiency as has been demonstrated [115,116], where pathway efficiency could be optimized by balancing the activity of the different components through adjusting the amount of the individual components (Figure 4a). Different scaffolds could potentially be combined in modular fashion, e.g., scaffolds for energy production and product synthesis could be added to the protocell during construction. Scaffolds might also assist in avoiding component incompatibilities resulting from employing catalytic pathways from different organisms in the same protocell. A more elaborate design could entail self-assembling nucleic acid scaffolds, engineered to enter the protocell through a nucleic acid channel (as described above) with spontaneous co-assembly of the scaffold and protein components. Current designs for artificial nucleic acid scaffolds are of a size that would be compatible with import in this manner if the import channels could be harnessed. 

Another plausible, and logical option when considering a protocell design based on living cells, is to isolate pathways and processes through internal membranes to compartmentalize the protocell, just as living cells use membrane encapsulated organelles for segregation of metabolic activities. This could take the form of an outer protocell membrane protecting internal protoorganelles that encapsulate individual pathways. Protoorganelles can be supplemented with other proteins and transporters if needed. Proof-of-concept has been demonstrated in a completely synthetic system [117] and in a hybrid synthetic-natural system utilizing microsomes as protoorganelles for assisted membrane protein expression [55]. Other compartmentalization solutions have utilized multi-compartment vesicle networks, essentially building individual chambers in a single protocell without the need for protoorganelles [56,118].

Additional design elements that could be considered to isolate specific processes could be the use of porous protein-based microcompartments derived from bacteria [119]. Such structures could serve both as scaffolds to support optimal organization of reaction components, and as barriers that compartmentalize the protocell interior and promote segregation of different activities. Alternatively simple chemical methods to promote phase separation of aqueous solutions could be implemented (see [120]) which have been observed to occur in solutions of biological macromolecules [121] and have been utilized to locally concentrate nucleic acids 3000 fold in artificial systems resulting in enhanced catalytic activity [122]. Compartmentalization by engineered phase separation may have utility in protocell design if it can be satisfactorily controlled in more complex systems. Although compartmentalization adds additional complexity it is relatively easy to accomplish through modified protocell design and assembly protocols, and the potential improvements in reaction efficiency and protocell function will likely outweigh the challenge of incorporating the additional components.

## 4. Microfluidic Production Lines for Protocell Construction

Increasingly, microfluidic devices are being utilized for the reproducible handling of pico- to nanoliter volumes. The advantages are that they are customizable, inexpensive, easy to manufacture and can be designed as modular systems with multiple functions integrated into a single device. The use of microfluidics in synthetic biology for design, optimization and component production has been recently reviewed [123,124]. Although no microfluidic workflow protocol for assembly of simple protocells has yet been described, it has the potential to provide a highly reproducible method for the manufacture, mixing and liposome encapsulation of components. Processes that could be undertaken using microfluidic devices, and of particular interest for protocell manufacture, are nucleic acid production, protein expression and purification, the functional encapsulation of internal components and liposome production. A miniature production and assembly line in the form of a self-contained microfluidic device should be capable of creating protocells from purified components with the high efficiency and reproducibility that can be obtained with microfluidics. While the design of a microfluidic device capable of assembling simple protein-based protocells is more complex than current devices, many of the individual design elements have been established and incorporated into existing microfluidic chip design.

### 4.1. Microfluidic Protein Production and Purification

**Figure 5:**
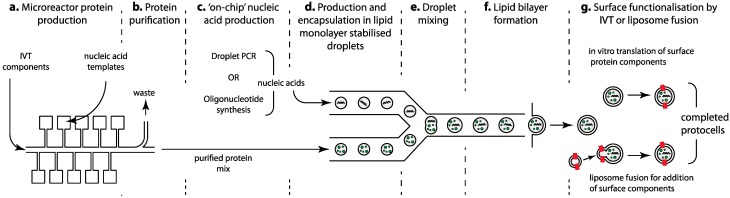
Microfluidic steps required for protocell assembly utilizing existing microfluidic production technology. (**a**) parallel production of the individual protein components in a microreactor array; (**b**) rapid purification of produced components; (**c**) production of nucleic acid components for “genomes” or scaffolding purposes by either PCR or oligonucleotide synthesis methods; (**d**) encapsulation of component mixes in lipid monolayer stabilized emulsions; (**e**) component mixing—multiple mixing steps with either symmetric or asymmetric droplets can be implemented; (**f**) formation of the outer bilayer leaflet around the emulsion droplets to complete the protocell membrane; (**g**) addition of surface receptors by liposome fusion or *in vitro* transcription/translation (refer to Figure 2 for more details of this stage of assembly). Note that not all alternatives for the individual steps are shown—refer to text for details.

Production of individual protein components can be carried out either by standard recombinant methods (off chip) or by *in vitro* transcription/translation within the constraints of a microfluidic device. An array of microreactors has been utilized for the parallel production of multiple (up to several hundred) proteins in a single device [125,126,127,128]. This design is ideal for the production of protocell protein components, which could potentially be all produced in parallel. A particularly promising example, in terms of application to protocells, is the use of immobilized nucleic acids as templates for *in vitro* protein synthesis [129,130,131]. Originally developed for protein microarray production (see [132] for details), immobilization of the nucleic acid templates localizes sites of protein production and can be utilized within a continuous flow of circulating transcription and translation reagents [133] resulting in protein production in individual reaction chambers (Figure 5a). Multiple affinity-tagged components could be produced simultaneously, with each chamber dedicated to production of one protein component, producing the entire complement of proteins in a single step. Extraction of the produced proteins from other components could be implemented with relative simplicity. Basic purification strategies have been achieved on a microfluidic device utilizing a continuous flow system where affinity tagged proteins can be subjected to a simple aqueous two-phase extraction [134]. Other strategies for on-chip purification have been described if additional steps are required to improve purity, e.g., Yu *et al.* [135], although currently these are only at the proof of concept stage, these strategies have potential (Figure 5b).

### 4.2. Microfluidic Production of Nucleic Acid “Genomes” and Scaffolds

The laboratory production of DNA is well established and if necessary nucleic acids can readily be produced “off-chip” for introduction into designs. It is however possible to create nucleic acids “on-chip” for direct incorporation into a microfluidic protocell production line. Strategies for nucleic acid production in microfluidic devices have been demonstrated largely as polymerase chain reaction (PCR)-based template replication and more recently as non-template driven oligonucleotide synthesis. While the “genome” (if required) for even a simple protocell would be too large to produce in a single piece, it could be split into multiple shorter fragments amenable to production. Nucleic acid scaffold components could readily be produced in the same system. A number of designs for microfluidic PCR devices have been put forward (these are beyond the scope of this review but have been reviewed previously [136]). These can be divided into continuous flow systems, where the reaction circulates through multiple temperature zones in a microfluidic chip, and single chamber devices, where the reaction is immobile and the chip cycles through the required temperatures. The former could utilize either a single aqueous phase or surfactant-stabilized droplets (droplet PCR), a more recent development that holds significant promise due to the encapsulation and isolation of the individual reactions [137,138,139,140] making the process amenable to droplet based methods for sorting and mixing described below. While there are additional complexities with PCR based methods as they require temperature cycling, both continuous flow and single-chamber microfluidics could be integrated with the production of other components for encapsulation if required. Alternatively the chemical synthesis of nucleic acids in microfluidic chips is now becoming a possibility with several designs recently described [141,142,143]. These allow the production of the required nucleic acids in the absence of any template requirement (Figure 5c).

### 4.3. Droplet Based Methods for Processing and Mixing

Microfluidic droplet-based technologies exploit the stability of surfactant-stabilized water in oil emulsions to compartmentalize miniature reaction volumes where components are stabilized in aqueous droplets as miniature microreactors. While emulsions have been utilized in the absence of microfluidics for encapsulating components in, for example *in vitro* evolution experiments [144], similar ideas have since been demonstrated in microfluidic devices [145,146,147]. Microfluidic designs provide exceptional control in the reproducible production and mixing of components that has previously not been achievable (recently reviewed in [148]). It is these qualities that make microfluidics particularly attractive for protocell construction (Figure 5d,e). While the stabilizing surfactant has predominantly been unsuitable for protocell production, the use of lipids as surfactants has been successfully demonstrated in microfluidic devices [149,150]. This technology has been applied to the functional encapsulation of enzymatic machinery [56,118] providing evidence that lipid-stabilized droplets offer the necessary biocompatible alternative suited to the initial stages of protocell construction.

### 4.4. The Use of Microfluidics for Liposome/Protocell Formation

Multiple designs for microfluidic devices for creating liposomes with potential as protocell membranes have been described (see reviews [151,152]). Although most existing methods for liposome production are based upon traditional hands-on approaches to GUV formation (described earlier), the enhanced control over production afforded by microfluidic devices has delivered unprecedented liposome reproducibility. The advent of microfluidics has facilitated development of new methods for liposome formation and component encapsulation. These notably include the use of double emulsions, allowing twin lipid monolayers to be brought together with an inter-leaflet oil phase such that removal of the residual oil creates the liposomes [153,154]. High encapsulation efficiency in the initial emulsions ensures high encapsulation in the liposomes. The latest and most promising developments stem from adapting this double emulsion technology to a continuous flow system, resulting in a rapid and reproducible method for liposome production [155,156] (Figure 5f). In these methods, the lipid-stabilized protocell emulsion prepared as described in the previous section becomes the precursor (or inner leaflet) of the protocell membrane. The encapsulation step can be immediately followed by fusion with an interfacial lipid monolayer, resulting in spontaneous assembly of the protocell membrane. The latter step provides the outer leaflet of the membrane in a process identical in principle to the more effective emulsion-based methods for GUV formation and encapsulation in bulk solutions (described earlier). An alternative method that may be of comparable utility is a membrane ejection, or pulsed jetting, approach to liposome formation [72,157,158], or a flow-driven process [159] where both leaflets of the protocell membrane are formed (as a unilamellar vesicle) in one step by ejection from a planar bilayer. This also allows for controlled encapsulation of components. 

Microfluidics has the capabilities to accommodate complex designs in a production line format. This might include formation of internal “protoorganelles” for segregating components or pathways, or simply creation of compartmentalized systems [56,118]. There are limits to the technology, however, and enthusiasm must be tempered with caution; too complicated an array of components may result in incompatibilities between assembly methods and sensitive biological reagents, e.g., denaturation.

Self-assembly can be achieved by either hands-on or microfluidic production, each holding its own technical challenges and limitations. The two are not mutually exclusive, however, and a prudent combination of case-by-case approaches is likely to enjoy the greatest success. 

## 5. Additional Considerations in Designing Purpose-Built Protocells

### 5.1. Internal Crowding

Living cells are characterized by a high degree of macromolecular crowding, where proteins occupy a significant amount of the available space. This undoubtedly influences cellular chemistry, such as the internal transmission of signals and the rate and efficiency of metabolic pathways. Increasing molecular crowding has been shown to result in increased gene expression in a simple synthetic transcription/translation system [160]. It may yet prove an important factor in protocell design. There are different ways crowding might be emulated, from a simple increase in the protein content (non-specific) to localized crowding on encapsulated scaffolds (specific) to tampering with the constitution of the aqueous “cytosol” with reagents such as polyethylene glycols. There are long-standing protocols for concentrating macromolecules and some simple microfluidic device designs [135] although combining concentration steps with addition of all the required components at suitable stoichiometric ratios may be a challenge to achieve successfully. It is likely to require a more sophisticated approach than simple mixing.

### 5.2. Protocell Lifetime

The lifespan of a particular purpose-built protocell depends on a number of factors: the stability of the individual components, whether there is any inbuilt capacity for renewal, whether reagents for biosynthesis and component renewal can be taken up and released across the membrane and so forth. The finite lifespans of simple protocells will be determined primarily by the stability of the component parts, in particular, the unfolding, non-specific aggregation, oxidation and degradation of proteins and the lipid membrane, and when utilized *in vivo*, the rate of clearance from the body.

It is possible to extend protocell lifespan by tactical means. Stabilizing the protein components should be relatively straightforward. Strategies could include using protein components derived from thermophilic bacteria or, if thermostable options are inaccessible, engineering proteins for improved thermostability, a technique explored in several biochemical studies [161,162,163,164,165,166,167,168,169]. New possibilities are continually emerging. For instance computational approaches aimed at engineering stability improvements, through identification of stabilizing mutations, prior to experimental verification [170,171,172] may have applicability to protocell design.

As the primary connection to the outside world, the bounding membrane of the protocell is particularly important. There are possibilities for stabilizing the structure of the membrane, many of which are derived from research into pharmaceutical and gene delivery vectors, that has for many years explored product encapsulation in liposomes (for reviews see [173,174,175]). A significant body of work is concerned with tailoring liposome surfaces for more desirable properties and improved stability, both *in vitro* and *in vivo*. *In vitro* stability can be improved by incorporation of chemical components, for example the complete substitution of biological lipids with synthetic polymer membranes [176], doping a lipid bilayer with polymerizable monomers to increase stability [177], or surface modification using saccharides [178], PEG [179] or other amphiphilic polymers [180]. There are extra parameters worth considering in protocells developed for *in vivo* usage (e.g., in protocells for drug delivery or production), where decreasing the rate of clearance from the body might be sufficient to achieve an increase in protocell lifetime or viability. Some design possibilities that decrease *in vivo* clearance have already been demonstrated in principle [179,181,182,183]. Crucially, integral membrane proteins can retain activity in some of these modified lipid systems [184,185,186].

### 5.3. Energy Generation

An energy source is essential in any cell, synthetic or living. The latter utilize biochemical respiratory pathways and the electron transfer chain to produce energy currency (e.g., adenosine triphosphate, ATP) to drive reactions. In the simplest protocell design, all of the required small molecule components including nucleotides and amino acids can be provided for biosynthesis, with ATP provided as an energy source [61]. These simple designs eliminate the need for energy generation and while impressive in their own right, are unlikely to be desirable designs due to the need for continual renewal of all molecules.

A more sustainable design would require suitable pathways for efficient energy generation and those necessary for amino acid and nucleotide synthesis, essentially creating a “self-sufficient” protocell capable of utilizing a readily available energy source such as sugars or sunlight to support function. Such a design would require a simple metabolic pathway to convert the “raw” energy into a usable form of chemical energy. A sophisticated implementation would be to segregate the energy-generating pathway in a protoorganelle, effectively mimicking the mitochondria and chloroplasts found in eukaryotic cells. In mitochondria and chloroplasts, the formation of a proton gradient is a central step in energy harvesting and conversion. While this may appear overly complicated, there is precedent in the form of artificial systems for ATP production. For example artificial chloroplasts utilizing ATP synthase and bacteriorhodopsin can be constructed from very few components [184]. In an elaborate self-assembling system, artificial proton pumps were engineered from modified cytochrome c and cytochrome c oxidase in artificial liposomes, or polymerosomes [187]. The proton pumps generated an artificial proton gradient across the polymerosome membrane, which could potentially be harnessed for energy production. Theoretically, it could be linked to the formation of ATP required in custom protocells to form the basis of a simple light driven energy source.

Metabolic pathways for burning simple sugars present another option. A complex artificial pathway utilizing glucose as a fuel source, consisting of 13 enzyme catalyzed steps, has been synthetically constructed [188]. Although not an encapsulated system, the demonstration that a functioning pathway of this complexity could be assembled is proof-of-concept of the strategy, and has implications for embedding biochemical pathways in constructing energetically self-sufficient protocells.

### 5.4. Ribosomes

Ribosomes are the living scaffold upon which proteins are translated from mRNA. Large enough to be seen under an electron microscope, they are a complex macromolecular assembly of proteins and nucleic acids, including ribozymes. They are an essential part of the catalytic machinery in any protocell where proteins will be produced. A strategy for the production of ribosomes and functional assembly from purified individual ribosomal protein components, and *in vitro* transcribed rRNA, was recently reported [189], representing a significant advance pertinent to ribosome incorporation into bottom-up protocells. Another option is extraction of affinity tagged ribosomes [190] for incorporation during protocell construction. 

### 5.5. Optimizing Design

Living organisms possess the unique ability to evolve in the face of selection pressures. Even unicellular organisms have this capability. Artificial protocells, in the absence of replication machinery, do not, as the ability to propagate is a necessity for evolution to occur. Under most circumstances the inability of protocells to evolve would be considered an advantage, preventing unwanted changes that diminish the design. The flip side of the coin is that protocells cannot respond to applied selection pressures to optimize performance. The optimal protocell must therefore be designed and pre-tested. 

Protocells will have a maze of functioning entities working in a restricted space and it would be naïve to assume that simply mixing together different parts will result in an optimally functional system. Component incompatibility is of major concern, where the requirement of one enzyme with respect to pH or salt composition will be very different to others requiring optimization of both the individual components and the entire design to ensure suitable activity. This is not an insurmountable issue however, as individual proteins can be modified using similar approaches to those outlined above for component stability improvements. Likewise compatibility and optimization of the individual metabolic pathways, while potentially difficult to resolve, could be assisted by computational (*in silico*) modeling. If the biophysical properties of the individual components have been established, this may be the simplest route to designing a functional protocell. In particular, it should assist with optimal component stoichiometry and activity of individual components for the desired pathway output. For example *in silico* methods have been used to optimize metabolic activity in synthetic bioreactors [191].

There are also possibilities for computational optimization of the entire protocell system, for example, computational methods have been successful in evaluating metabolism in complex organisms. A case in point is the now well established genome scale model of metabolism in *E. coli*, first described in 2000 [192] and significantly expanded since [193,194,195]. Similar metabolic models for other organisms have been described including *Saccharomyces cerevisiae* [196], *Pseudomonas putida* [197], and even *Homo sapiens* [198]. These organisms are many times more complex than protocell designs. In principle, it would be expected that the far simpler protocell designs would be amenable to verification and optimization *in silico*.

High throughput experimentation also has a place in design optimization, and microfluidic droplet based approaches could be utilized in the directed evolution of individual components prior to design completion [145,146,147].

### 5.6. Gene Regulation

In purpose-built protocells containing a “genome”, simple gene regulatory mechanisms borrowed from nature may be of use in controlling gene transcription processes. While transcriptional control is exceptionally complex in living organisms (see [199]), this is unnecessary in protocells and control could amount to grouping enzymes or biochemical pathways into sets that can be generically controlled by one or more transcription factors. 

Custom engineered transcription factors have been used to effectively regulate gene expression in cellular systems allowing targeting and regulation of transcription from specific DNA sequences. Classes of transcription factors that have been shown to be amenable to custom modification include the zinc finger family of transcription factors [200,201,202] and the transcription activator-like effector transcription factors [203,204]. The basic design principles for DNA binding of zinc finger transcription factors have been described [205,206,207,208] opening up transcription factor design as a viable method for gene regulation in artificial systems. The principles of designing transcription factors such as these could be harnessed with relative ease in protocell construction as all components (transcription factors and DNA sequences for transcription factor binding) could be built as a functioning unit.

An alternative and simpler option to regulate protocell gene expression is stoichiometric addition of gene copy number to control the relative levels of individual components, without more complex regulation. Increasing copy number of individual components increases expression levels of some components relative to others. Although not true regulation, this would result in some control of the production of individual components at the design stage. This method has been utilized in metabolic engineering applications in combination with other strategies [15], and could be applicable in simple protocell designs.

Engineered oscillator circuits, where the activity of the system follows a predetermined amplitude and period as a result of feedback loops, are also likely to have an important role in protocell gene expression and catalytic systems. Examples of enzymatic oscillators [209] and genetic oscillator circuits [133,210,211] have been demonstrated in principle. Circuits such as these are likely to play an important role in the control of protocell gene expression and metabolism.

Small volume systems have additional design considerations, as the process of partitioning small volumes of protocell components results in a stochastic distribution affecting the biochemical properties of the system. This has been observed in the gene expression of both living cells [212] and artificial biochemical oscillator designs where partitioning into femtoliter volume compartments results in significant variation in the observed oscillation periods and amplitudes [210]. Volumes of this size are in the range required for protocell design, therefore robust designs that either eliminate this variability or utilize it to an advantage are likely to be required.

## 6. Conclusions

Building a complex self-assembling nanomachine from a collection of diverse biological components would be a significant scientific achievement, but one that has not yet been realized. Although many of the design principles have been established and there is precedent for some of the important componentry to be successfully reconstituted *in vitro*, attaining sufficient knowledge to effectively utilize the components in construction of a viable protocell represents a substantial undertaking. The task may be significantly greater than has yet been accomplished in characterizing the proteins in a native environment. Nevertheless, the potential benefits make the effort more than worthwhile. The simplicity, non-autonomous nature and finite lifespan of non-replicative protocells relative to living organisms holds many potential applications for synthetic biology and molecular medicine.

Part of a good design remit is to choose a minimal system that will function as required. Protocells with well-defined synthetic applications, such as fuel or pharmaceutical synthesis, could be designed as stripped-down models, incorporating essential biosynthetic and bioenergetic pathways, but eliminating a multitude of other complex processes required for “life”. Another means of simplification is inherent in the separation between the protocell and its construction process. Not everything has to be encapsulated. Offloading some key functions, for example through component production in a microfluidic production line and self assembly via engineered scaffolds, would result in protocells of significantly reduced complexity that will still be capable of carrying out a designated function. Many designs reported to date are tantalizingly close to the complexity required for an elementary functioning protocell. The use of technologies that have been evaluated and implemented *in vitro* to a prototype stage, in combination with a range of components that have been functionally reconstituted, also *in vitro*, will serve as the basis of useful protocells where the finite lifetime is determined only by the longevity of the components.

There is significant ground to be covered before functional protocells become a reality. As well as basic construction issues, design will focus on maintaining compatibility—whether by separation of processes or tailoring of reagents. Steady progress in the area has still not yet advanced to the point where a fully functional self-replicating cell can be built. Many would consider the major stalling points to be capabilities for self-replication and division. Whilst impressive steps have been made towards this goal, it is likely to be quite some time before a true autonomous artificial cell can be realized, whereas the design and construction of simpler machines built for a vast array of important synthetic or bio-delivery purposes is almost within grasp.
